# PpFab: An efficient promoter toolkit in *Physcomitrium Patens*

**DOI:** 10.1093/plphys/kiae332

**Published:** 2024-06-12

**Authors:** Guangyu Luo, Hao Ye, Mengxuan Xu, Xiaofang Li, Jianxuan Zhu, Junbiao Dai

**Affiliations:** Shenzhen Branch, Guangdong Laboratory for Lingnan Modern Agriculture, Shenzhen Key Laboratory of Agricultural Synthetic Biology, Genome Analysis Laboratory of the Ministry of Agriculture and Rural Affairs, Agricultural Genomics Institute at Shenzhen, Chinese Academy of Agricultural Sciences, Shenzhen 518124, China; Shenzhen Key Laboratory of Synthetic Genomics, Guangdong Provincial Key Laboratory of Synthetic Genomics, Shenzhen Institute of Synthetic Biology, Shenzhen Institute of Advanced Technology, Chinese Academy of Sciences, Shenzhen 518055, China; Shenzhen Branch, Guangdong Laboratory for Lingnan Modern Agriculture, Shenzhen Key Laboratory of Agricultural Synthetic Biology, Genome Analysis Laboratory of the Ministry of Agriculture and Rural Affairs, Agricultural Genomics Institute at Shenzhen, Chinese Academy of Agricultural Sciences, Shenzhen 518124, China; Shenzhen Branch, Guangdong Laboratory for Lingnan Modern Agriculture, Shenzhen Key Laboratory of Agricultural Synthetic Biology, Genome Analysis Laboratory of the Ministry of Agriculture and Rural Affairs, Agricultural Genomics Institute at Shenzhen, Chinese Academy of Agricultural Sciences, Shenzhen 518124, China; Shenzhen Key Laboratory of Synthetic Genomics, Guangdong Provincial Key Laboratory of Synthetic Genomics, Shenzhen Institute of Synthetic Biology, Shenzhen Institute of Advanced Technology, Chinese Academy of Sciences, Shenzhen 518055, China; Shenzhen Branch, Guangdong Laboratory for Lingnan Modern Agriculture, Shenzhen Key Laboratory of Agricultural Synthetic Biology, Genome Analysis Laboratory of the Ministry of Agriculture and Rural Affairs, Agricultural Genomics Institute at Shenzhen, Chinese Academy of Agricultural Sciences, Shenzhen 518124, China; Shenzhen Branch, Guangdong Laboratory for Lingnan Modern Agriculture, Shenzhen Key Laboratory of Agricultural Synthetic Biology, Genome Analysis Laboratory of the Ministry of Agriculture and Rural Affairs, Agricultural Genomics Institute at Shenzhen, Chinese Academy of Agricultural Sciences, Shenzhen 518124, China; Shenzhen Branch, Guangdong Laboratory for Lingnan Modern Agriculture, Shenzhen Key Laboratory of Agricultural Synthetic Biology, Genome Analysis Laboratory of the Ministry of Agriculture and Rural Affairs, Agricultural Genomics Institute at Shenzhen, Chinese Academy of Agricultural Sciences, Shenzhen 518124, China; Shenzhen Key Laboratory of Synthetic Genomics, Guangdong Provincial Key Laboratory of Synthetic Genomics, Shenzhen Institute of Synthetic Biology, Shenzhen Institute of Advanced Technology, Chinese Academy of Sciences, Shenzhen 518055, China

Dear Editor,

Many metabolic pathways for the synthesis of natural products require multiple genes that are regulated by specific regulatory networks. Within the field of synthetic biology, the limitations of common chassis cells, such as yeast and bacteria, have prompted exploration of plant chassis cells due to their unique advantages (Cravens et al. 2019; Lin et al. 2023). *Physcomitrium patens* (*P. patens*), a unique model plant with high homologous recombination efficiency, holds promise as a plant chassis in synthetic biology (Reski et al. 2018; Rensing et al. 2020; Roell and Zurbriggen 2020). Urgent research is required for the characterization of gene element libraries and the establishment of a multigene expression system. Previous studies emphasized the importance of promoters in gene expression regulation and highlighted *P. patens*’ adaptability to heterologous promoters (Horstmann et al. 2004; Niederau et al. 2024), laying a theoretical foundation for the subsequent characterization of diverse promoters. Additionally, pioneering study has demonstrated the feasibility of an inducible gene expression system in *P. patens* (Kubo et al. 2013). However, investigations regarding the regulation of the expression of multiple genes within metabolic pathways are still lacking. Furthermore, the cataloging and standardization of biological elements, particularly promoters, in *P. patens*, are far from complete (Peramuna et al. 2018). In this study, we utilized a dual fluorescence reporter system (DFRS) to characterize the transcriptional activity of different promoters in *P. patens*. Expanding on the YeastFab assembly, a high-throughput method for genetic parts construction in yeast cells (Guo et al. 2015; Garcia-Ruiz et al. 2018; Malcı et al. 2022), and incorporating the betalain reporter system (RUBY) (He et al. 2020; Yu et al. 2023), we established the *P. patens* Fab (PpFab) platform. The PpFab platform is a convenient and intuitive platform for detecting the effects of different promoter and gene combinations in the metabolic pathway on the yield of end products.

To ensure a precise and swift evaluation of the transcriptional activity of chosen promoters, our study acts as a preliminary experiment for high-throughput analysis. We utilized an in vivo transient DFRS employing luciferase (LUC) as a reporter gene and β-glucuronidase as the internal reference, expressed in the *P. patens* (Hackenberg et al. 2015) (Fig. 1A). The promoter to be tested was integrated into the DFRS by fusing with *LUC*, and the transcriptional activities were determined by measuring the relative activities of LUC. This system was validated by promoters, as illustrated in Fig. 1B and Supplementary Fig. S1A. The results demonstrated that these widely used plant promoters and DFRS functioned effectively in *P. patens*.

**Figure 1.**
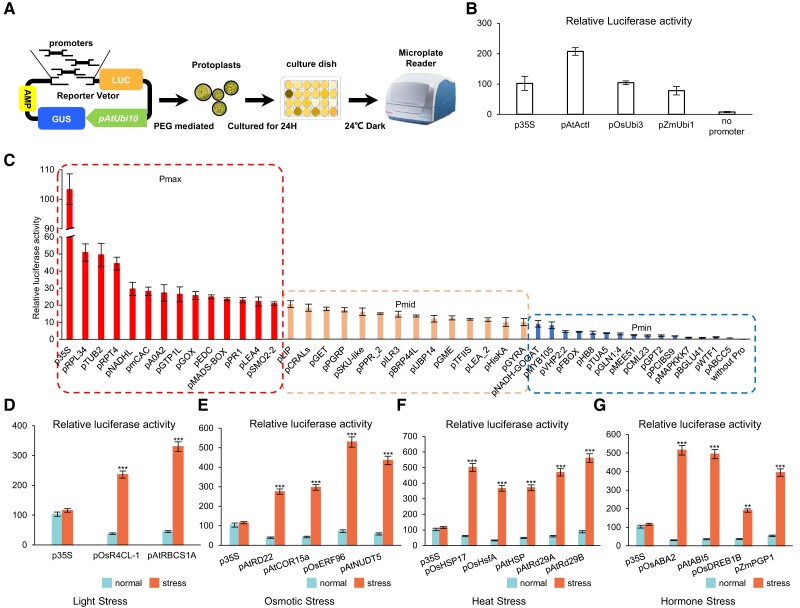
Characterization of native promoters using a DFRS. **A)** Each promoter was inserted upstream of the LUC gene in a plasmid, which also contains a GUS fluorescent protein driven by the *AtUBI10* promoter and NOS terminator. AMP, ampicillin resistance gene; GUS, β-glucuronidase report gene; LUC, luciferase report gene; PAtUbi10, the promoter sequences of *AtUbi10.***B)** Detection of plant constitutive and endogenous promoter activity. Promoters-LUC served as the reporter for both plant constitutive and endogenous promoter activity, with AtUBI10-GUS as the internal control. **C)** Detection of endogenous promoter activity in *P. patens* by the DFRS. (Define relative transcriptional activity above 20 as Pmax, 20 to 10 as Pmid, and less than 10 as Pmin.). **D)** Detection of frequent exogenously induced promoters (light related) activity in *P. patens* by the DFRS. **E)** Detection of exogenous promoters (osmotic related) activity in *P. patens* by the DFRS. **F)** Detection of frequent exogenously induced promoters (temperature-related) activity in *P. patens* by the DFRS. **G)** Detection of exogenous promoters (hormone related) activity in *P. patens* by the DFRS. Promoters-LUC was used as the reporter. *AtUBI10*-GUS was used as the internal control. The data in panels **(C)** to **(E)** are presented as means ± Sd. Asterisks indicate a significant difference compared to wild type (WT) (two-way ANOVA: *P < 0.05, **P < 0.01, ***P < 0.001). At least three independent biological replicates were performed with similar results.

Using this platform, we characterized a large number of endogenous promoters from RNA-seq data (BioProjects PRJNA294412, PRJNA601618) (Stevenson et al. 2016; Haas et al. 2020), in addition to promoters of common model plants. The results revealed a range of promoters with variable transcriptional strengths, with the majority being moderately weak and only a few displaying strong activity (Fig. 1C). Promoters were categorized into three groups based on their transcriptional activities: Pmax (>20), Pmid (10 to 20), or Pmin (<10). The transcriptional activities tested in the results irregularly differ from those in the RNA-seq data, possibly due to alternations in the neighboring genomic context, transitions between species, or changes in the landscape of gene expression (Puthiyaveetil et al. 2021; Villao-Uzho et al. 2023).

In addition, to assess whether inducible promoters could be accurately characterized in this system, we introduced serval model plant adversity-responsive promoters and subjected them to various treatments, including darkness, osmotic (10% W/V PEG), heat stress (37 °C), and hormone (0.5 *μ*m ABA). The results showed that a variety of inducible promoters can be effectively characterized in vivo by this system, exhibiting correct responses to various inductions (Fig. 1, D to G).

Through the characterization of a variety of endogenous promoters by the DRFS system in *P. patens*, we found that the core promoter elements of endogenous promoters in *P. patens*, similar to other species, are essential for maintaining their transcriptional activity. Furthermore, we found that they synergistically regulate the transcriptional activity of the promoter along with the upstream regulatory elements (Supplementary Fig. S1B). Subsequent experiments involving the replacement of *cis*-acting elements in the constitutive promoter 35S and the hormone-inducible promoter pD2 *OsRAB21* (Os11g0454300) showed significant changes in transcriptional activity (Supplementary Fig. S1, C and D), suggesting the potential applicability of the identified *cis*-acting element regulatory mechanism to *P. patens*, significantly enriching the existing promoter library.

In our investigation of the most effective promoter combinations, we integrated the RUBY system with the YeastFab system, establishing a high-throughput and visually intuitive strategy for assembling promoters to enhance metabolite synthesis. This approach led to the development of an efficient metabolite assembly system customized for *P. patens*, named PpFab (Fig. 2A). To verify the function of the RUBY system in *P. patens*, the three RUBY genes were sequentially driven by the promoters of *PpACTIN3*, *CaMV35S,* or without a promoter. Betalain were analyzed by HPLC. The results showed variations among the products of the betalain (Fig. 2B), confirming the effectiveness of the RUBY and PpFab systems in *P. patens*. Meanwhile, the visualized red color intensity of the leaves measures the strength of the effect of promoter combinations on metabolite synthesis (Fig. 2C).

**Figure 2.**
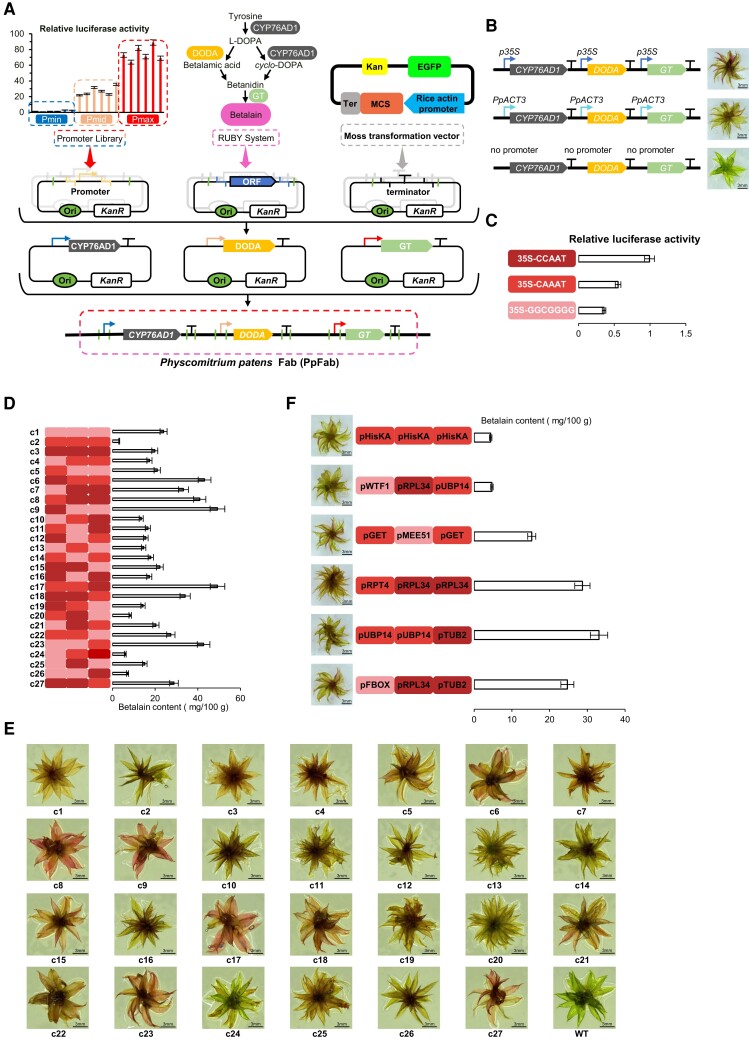
Establishment of the PpFab stable reporter system and optimization of transcriptional combinations of genes in the metabolic synthesis pathway (PpFab). **A)** The development of the PpFab report system. The three genes of the RUBY system were used as the three transcriptional units (TUs), and then the promoter sequences in each TUs were replaced using the promoters characterized in the promoter libraries. The terminator sequences in each TUs were replaced using the moss transformation vector (pTFH22.4) which were then assembled into PpFab. **B)** The recombinant plants transformed by connecting the promoter of *PpACTIN3* and *CaMV35S* and no promoter sequence into the PpFab reporter system will produce different degrees of red. The p35S, pACT3 promoters and no promoter sequence used to replace all three promoters of the RUBY for the PpFab system in *P. patens*. **C)** The transcriptional activity of the 35S promoter containing different enhancers (35S-GGCGGGG, 35S-CAAAT, 35S-CCAAT) measured by DFRS. **D)** By random combinations of three selected promoters, we obtained 27 combinations of metabolic pathways with different betalain synthesis capacities. which transplanted into *P. patens*, and the betalain content in each plant was assayed by HPLC. **E)** The phenotypes of color for the recombinant *P. patens* plants transformed by random promoter combinations. **F)** Characterization of combinations of six metabolites with varying yields by using the promoter library of *P. patens*, which in turn modulates the final synthetic yield of betalain. The data in panels **(C)** to **(E)** are presented as means ± Sd. Asterisks indicate a significant difference compared to WT. At least three independent biological replicates were performed with similar results.

Subsequently, we selected three promoters (35S-GGCGGGG, 35S-CAAAT, and 35S-CCAAT), corresponding to weak (P1), medium (P2), and strong promoters (P3), respectively (Fig. 2D). These promoters were systematically paired with three betalain genes from the RUBY systems, resulting in a total of 27 combinations (Fig. 2E). The result illustrates representative phenotypic features, where the red color served as a visual indicator for beet red pigment production, exhibiting a notable correlation with HPLC quantitative results (Fig. 2, E and F). The findings underscore the necessity for careful optimization within the biosynthetic pathway, emphasizing that achieving high final synthesis product levels requires more than just the insertion of each relevant gene with a strong promoter.

To validate this, we paired characterized promoters with three RUBY genes in diverse combinations to assess betalain production levels. Fig. 2, D and E display phenotypes with visible red color accumulation corresponding to their betalain production, as examined by HPLC. These results align with the earlier findings from the 27 combinations (Fig. 2E), affirming the reliability of the PpFab system and our screening strategy. The results also highlight the guiding role of PpFab in optimizing the biosynthetic pathway in *P. patens*, showcasing its ability to enhance product synthesis through the optimal selection of promoter combinations within the chosen pathway.

In summary, we devised PpFab and integrated it with DFRS and RUBY, establishing a robust strategy for genetic part selection and functional gene detection in *P. patens*. Through this cohesive approach, we accomplished the following: (1) Created an extensive promoter library comprising a range of characterized endogenous and exogenous promoters, as well as synthetic promoters with specific modifications. (2) Enhanced the arrangement and combination of multiple genes and promoters, thereby enabling efficient metabolite synthesis in *P. patens*. This integrated platform offers a significant repository of gene components and strategies for synthesizing metabolic pathways, laying the groundwork for establishing a biosynthesis factory in *P. patens*. Additionally, it contributes to the development of an extensive promoter library for synthetic biology applications in the field.

## Author contributions

J.D. conceived and supervised the research. G.L., M.X., X.L., and J.Z. performed the experiments. G.L., H.Y., and J.D. analyzed the data and wrote the manuscript. All authors read and approved the final manuscript.

## Supplementary data

The following materials are available in the online version of this article.

**Supplementary Figure S1.** Functional element deletion or replacement analysis of endogenous promoters.

**Supplementary Data Set 1**. The genes and sequences used in promoter library by DFRS.

**Supplementary Data Set 2**. The exogenous genes and sequences used for induced promoters by DFRS.

**Supplementary Data Set 3.** The sequences for the modification of *cis*-acting elements by constitutive promoter (p35S) and induced promoters (pD2) used for DFRS.

## Supplementary Material

kiae332_Supplementary_Data
