# Supplementary material for: PpFab: An efficient promoter toolkit in Physcomitrium Patens
Source: Plant Physiol. 2024 Jun 12;196(1):2–6. doi: 10.1093/plphys/kiae332 (PMC11376402; doi:10.1093/plphys/kiae332)
Supplement: kiae332_Supplementary_Data [file kiae332_supplementary_data.zip › Supplemental Figure.pdf]

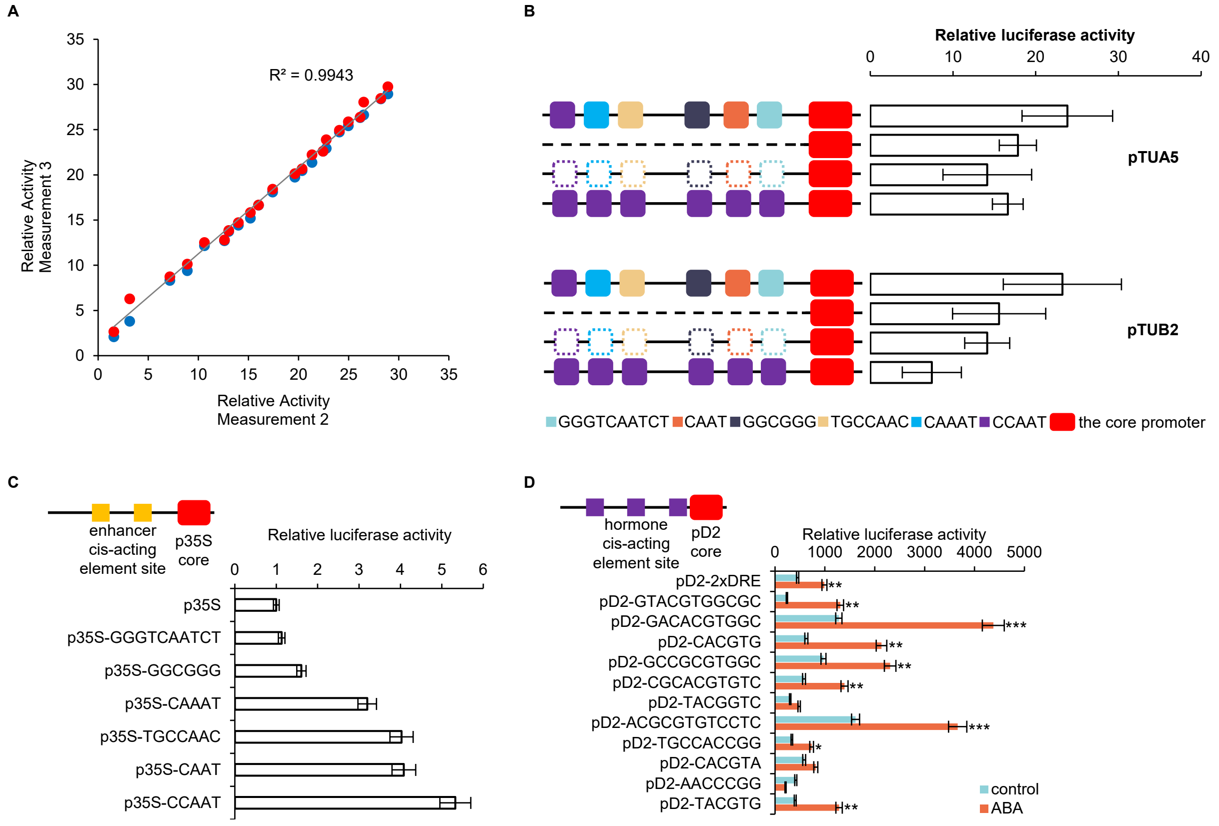

Figure S1. Functional element deletion or replacement analysis of endogenous promoters.

(A) High correlation between repeated measurements (R^2 = 0.99). Each dot represents a promoter, which was measured in two independent experiments. **(B)** Structural diagram of endogenous promoter and its functional elements deletion or replacement. The different colors represent different cis-acting elements and the core promoter. The cis-acting elements referred to here are the GC-box and the CAAT-box. **(C)** Effects of functional element deletion or substitution on endogenous promoter function. LUC and GUS activity was measured in 3 independent transformants for each construct and was shown as average values. The colored squares represent enhancer cis-acting elements contained in the promoter sequence. **(D)** Using pD2-2xDRE as the template, the two DRE and one ABRE sites of pD2-2xDRE were replaced by ABA-responsive cis-acting element with different compositions. The colored squares represent by hormone cis-acting elements contained in the promoter sequence. The data in panels C-E are presented as means± SD. Asterisks indicate a significant difference compared to wild type (WT) (two- way ANOVA: * $P< 0.05$, ** $P< 0.01$, *** $P< 0.001$). At least three independent biological replicates were performed with similar results.
