# Supplementary material for: PpFab: An efficient promoter toolkit in Physcomitrium Patens
Source: Plant Physiol. 2024 Jun 12;196(1):2–6. doi: 10.1093/plphys/kiae332 (PMC11376402; doi:10.1093/plphys/kiae332)
Supplement: kiae332_Supplementary_Data [file kiae332_supplementary_data.zip › Supplemental Material and MethodsClean version.docx]

**Material and Methods**

**Plant materials and growth conditions.**

Wild-type *Physcomitrium patens* (*P. patens*) or recombinant lines created in the Gransden WT (*P. patens*) background were propagated in growth chambers at 25℃ constant temperatures, respectively, with a relative humidity of 50% to 60%, and light and dark periods of 16 h and 8 h, respectively, before treatment. Fresh protonema cultivated on BCDAT agar medium. The BCDAT medium was composed of 1.84 mM KH_2_PO_4_ (pH 6.5), 10 mM KNO_3_, 1 mM MgSO_4_, 45 μM FeSO_4_, trace element solution (10 μM H_3_BO_3_, 0.22 μM CuSO_4_, 2 μM MnCl_2_, 0.1 μM Na_2_MoO_4_, 0.19 μM ZnSO_4_, 0.23 μM CoCl_2_ and 0.17 μM KI), 1 mM CaCl_2_, 5 mM ammonium tartrate and 0.7% agar. And fresh gametophore cultivated on BCD agar medium (BCDAT medium without ammonium tartrate).

21-day-old gametophore grown in the growth chambers is for the 48 H stress treatment. For osmotic stress construction, the gametophore was treated at with 200 mM D-mannitol; the double light intensity was treated for light stress experiment, the 37 ℃ is for the heat stress ,and the 50 μM ABA treatment is for the hormone stress experiment.

**Endogenous promoter screening principle.**

Using the previous transcriptome data, all genes were sorted according to logCPM in different treatments, and all genes with logCPM values greater than 10, 10-7.5, 7.5-5, 5-2.5, 2.5-0 bands were selected respectively and sorted again according to PValue, and the three genes with the largest, smallest, and closest to 0 FC values were selected as alternative genes.

**Vector construction and reorganization.**

**DFRS vector:** The region of 1,500 bp upstream for the selected genes were chosen as the promoter sequence of the genes, which were synthesized and then ligated into the front of the luciferase (Brasier and Ron, 1992) by using BamHI and Ncol has been used as a transient assay for transcriptional activity.

**PpFab vectors:** The selected promoters were ligated into the front of *CYP, DODA,* and *GT* in the PpFab system using BsmBI. The vectors were linearized using NotI. The target fragment was homologously recombined using the sequence of *PpHEX* as the homologous arm and integrated into the corresponding position of the genome *Chr2*.

**Transformation of *P. patens* and acquisition of recombinant plants.**

The *P. patens* protonema culture protocols have been described in detail above. In brief, BCDAT agar medium (BCDAT stands for stock solutions B, C, D, and ammonium tartrate) was used for regular *P. patens* culture at 24 °C with a relative humidity of 50% to 60%, and light and dark periods of 16 h and 8 h.

The transformation was performed using protoplasts. 7-day-old protonema was incubated in the solution containing 2% (w/v) driselase and 8% (w/v) mannitol at room temperature in a dark light for 30 min with gentle rocking. The solution was filtered using 50 μm nylon-mesh and centrifuged at 120 g for 3 min to collect protoplasts. The precipitation of protoplasts was washed twice in 20 ml 8% mannitol. Protoplast concentration before transformation was adjusted to 1.6 × 10^6^ ml^−1^ by adding MMM solution (0.1 % MES pH 5.6, 15 mM MgCl_2_, 9.1% (w/v) mannitol). 300 μL of protoplast solution were mixed with 30 μL of Linear DNAs (20 μg) for 15 min at room temperature, then 330 μL of 40% (w/v) polyethylene glycol solution (10 mM Tris-HCl pH 8.0, 100 mM Ca(NO_3_)_2_, 8% mannitol) was incubated for 30 min. The protoplast followed by incubation with W5 solution (pH 5.7) (154 mM NaCl, 125 mM CaCl_2_, 5 mM KCl, 2 mM MES) in the dark at 25 °C overnight. For recombinant plant the protoplasts were spread on PRMB agar medium lined with sterile cellophane. After three days, the cellophane containing regenerated protoplasts was transferred to BCDAT medium containing antibiotics for screening. Positive seedlings were allowed to grow before genotyping PCR or sequencing to confirm whether the target fragment was integrated into the genome.

We generated 27 PpFab recombinant plants with all combinations based on random arrangements of promoters with three promoters (35S-GGCGGGG, 35S-CAAAT, and 35S-CAAT) with different strengths. These plants were subsequently categorized as "high," "medium," or "low" according to their betalain content. Next, we calculated the average number for each group and selected the two combinations closest to each average, resulting in a total of 6 combinations. Based on these 6 combination patterns, we randomly selected promoters from the library for testing according to promoter categories (Pmax, Pmid or Pmin). We performed vector construction and obtained recombinant plants, which were then finally assayed for betalain content.

**Luciferase and GUS Activity Assay.**

The transformed protoplasts were recovered in culture for 12 H and lysed using a protoplast lysis buffer (2.5 mM Trisphosphate, pH 7.8, 1 mM DTT, 2 mM DACTAA, 10% (v/v) glycerol, 1% (v/v) TritonX-100.), taking 20 µl of protoplast lysates were mixed with 100 µl of LUC mix (Promega). LUC activity was measured using the microplate reader with chemiluminescence. Meanwhile take 2 µl of protoplast lysates were mixed with 10 µl of GUS mix (10 mM Tris-HCl, pH 8.0, 1 mM MUG (4-methylumbelliferyl β-D-glucuronide; Gold Biotechnology), 2 mM MgCl_2_). After the mixture was kept for 30 min at 37 ℃, 100 µl of 0.2 M Na_2_CO_3_ was added, and GUS activity was measured using the plate reader with the excitation filter at 355 nm and the emission filter at 460 nm.

**High performance liquid chromatography (HPLC).**

Take 1 g of the transformed *P. patens* grind by liquid nitrogen, added to 50 ml 95% ethanol, centrifuge at 3000 rmp for 20 mins and extracted the supernatant, then filtered through 0.45 μm filter membrane and 20μl was taken as sample. The chromatographic column was a Symmetry C18 ®5 μm (4.6X150 mm) column with the mobile phases of A: methanol, B: water-glacial acetic acid (A:B=20:80) at a flow rate of 1.0 ml/min, and the column temperature was 25 ℃. The detection wavelength was 535nm.
